# The risk of intra-urethral Foley catheter balloon inflation in spinal cord-injured patients: Lessons learned from a retrospective case series

**DOI:** 10.1186/s13037-016-0101-1

**Published:** 2016-05-21

**Authors:** Vaidyanathan Subramanian, Bakul M. Soni, Peter L. Hughes, Gurpreet Singh, Tun Oo

**Affiliations:** Regional Spinal Injuries Centre, Southport and Formby District General Hospital, Town Lane, Southport, PR8 6PN, UK; Department of Radiology, Southport and Formby District General Hospital, Town Lane, Southport, PR8 6PN, UK; Department of Urology, Southport and Formby District General Hospital, Town Lane, Southport, PR8 6PN, UK

**Keywords:** Spinal cord injury, Foley catheter, Urethral catheterisation

## Abstract

**Background:**

Inflating the balloon of Foley catheter in urethra is a complication of urethral catheterisation. We report five patients in whom this complication occurred because of unskilled catheterisation. Due to lack of awareness, the problem was not recognised promptly and patients came to harm.

**Case series:**

1. A tetraplegic patient developed pain in lower abdomen and became unwell after transurethral catheterisation. CT pelvis revealed full bladder with balloon of Foley catheter in dilated urethra.
2. Routine ultrasound examination in an asymptomatic tetraplegic patient with urethral catheter drainage, revealed Foley balloon in the urethra. He was advised to get catheterisations done by senior health professionals.
3. A paraplegic patient developed bleeding and bypassing after transurethral catheterisation. X-ray revealed Foley balloon in urethra; urethral catheter was changed ensuring its correct placement in urinary bladder. Subsequently, balloon of Foley catheter was inflated in urethra several times by community nurses, which resulted in erosion of bulbous urethra and urinary fistula. Suprapubic cystostomy was performed.
4. A tetraplegic patient developed sweating and increased spasms following urethral catheterisations. CT of abdomen revealed distended bladder with the balloon of Foley catheter located in urethra. Flexible cystoscopy and transurethral catheterisation over a guide-wire were performed. Patient noticed decrease in sweating and spasms.
5. A paraplegic patient developed lower abdominal pain and nausea following catheterisation. CT abdomen revealed bilateral hydronephrosis and hydroureter and Foley balloon located in urethra. Urehral catheterisation was performed over a guide-wire after cystoscopy. Subsequently suprapubic cystostomy was done.

**Conclusion:**

Spinal cord injury patients are at increased risk for intra-urethral Foley catheter balloon inflation because of lack of sensation in urethra, urethral sphincter spasm, and false passage due to previous urethral trauma. Education and training of doctors and nurses in proper technique of catheterisation in spinal cord injury patients is vital to prevent intra-urethral inflation of Foley catheter balloon. If a spinal cord injury patient develops bypassing or symptoms of autonomic dysreflexia following catheterisation, incorrect placement of urethral catheter should be suspected.

## Introduction

In 2010, we proposed that incorrect placement of a Foley catheter leading to inflation of Foley balloon in urethra in a spinal cord injury patient should be declared as a “never event” [1]. Although several advances have been made in management of spinal cord injury patients during this decade, we continue to see this complication of Foley catheter balloon inflation in urethra in spinal cord injury patients. We present five patients, in whom we detected this complication between 2012 and 2015 when these patients visited spinal injuries centre; it is possible that some other patients had developed this complication which went either undetected in the community or managed symptomatically in general hospitals without being recognised. The aim of this report is to raise awareness among health professionals in order to prevent intra-urethral Foley catheter balloon inflation in spinal cord injury patients and thereby reduce harm to patients.

## Case presentation

### Case 1

A 61-year-old male patient with tetraplegia attended a hospital as he was bypassing the indwelling urethral catheter. Transurethral catheterisation was performed by a health professional in the community. But, the catheter did not drain urine satisfactorily. The patient developed severe pain in lower abdomen; subsequently, he started getting pain in both kidneys as well; he developed loss of appetite and felt sick. He came to spinal unit after two weeks. On clinical examination, a long segment of Foley catheter was found to be lying outside penis. The patient was clear that the same length of catheter was lying outside his penis right from the time of insertion. Blood tests: C-reactive protein: 222.4 mg/L; urea: 1.8 mmol/L; Creatinine: 39umol/L. Clinical diagnosis was misplacement of urinary catheter in urethra with urosepsis.

**Fig. 1:**
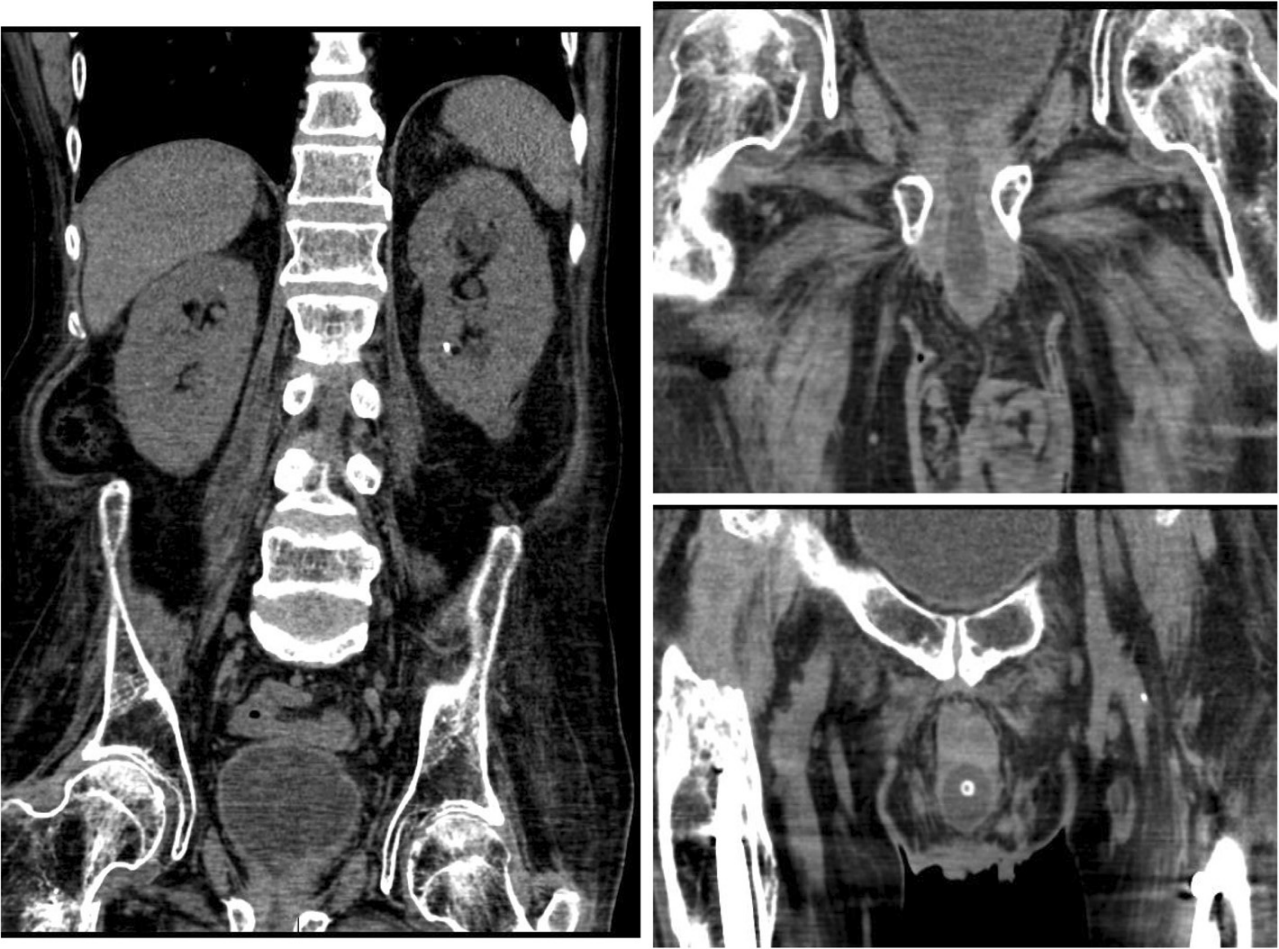
Case 1. Left panel: Coronal section of non-contrast CT of abdomen revealed full bladder; Foley catheter was not seen within the urinary bladder. Right Top panel: Coronal section of CT of pelvis showed fluid distension of the membranous and prostatic urethra. Foley catheter was not seen in bladder or in proximal urethra. Right bottom panel: Coronal section of CT of pelvis revealed balloon of Foley catheter in the proximal penile urethra

CT of pelvis was requested to locate the position of the balloon of Foley catheter. However, CT of abdomen was performed, which revealed full bladder. Foley catheter was not seen within the urinary bladder (Fig. 1 Left panel). This scan did not include pelvis; therefore precise location of the Foley balloon could not be ascertained. The patient was sent to radiology department again for scan of pelvis. CT of pelvis revealed fluid distension of the membranous and prostatic urethra (Fig. 1 Right Top panel); the balloon of Foley catheter was located in the proximal penile urethra (Fig. 1 Right Bottom panel).

Urethral catheter was removed; a Foley catheter with 20 ml balloon was inserted in the urinary bladder as a 10 ml balloon might slip through dilated bladder neck into urethra; turbid urine was drained. This patient was prescribed 160 mg of gentamicin followed by Ciprofloxacin by mouth. Three days later, the catheter got blocked; a district nurse changed the catheter; but inserted a Foley catheter with 10 ml balloon. The catheter did not drain urine satisfactorily and the patient came to spinal unit. On clinical examination, it was obvious that the catheter had been misplaced in the urethra, either got curled itself in dilated urethra during insertion or slipped into urethra subsequently. The Foley catheter had a radio-opaque marker; therefore, X-ray of pelvis was taken to find out precise location of the tip of the catheter. X-ray of pelvis revealed the tip of Foley catheter in the urethra. The catheter was removed and a Foley catheter with 20 ml balloon was inserted. The patient was reviewed after eleven days. The catheter was draining fine and patient was well.

### Case 2

A 66-year-old, British Caucasian male, sustained hyperextension injury to neck in a bicycle accident in 2011. He developed tetraplegia at C-5. This patient had been managing his bladder by indwelling urethral catheter. Urethral catheter was being changed by district nurses. About 29 months after spinal injury, this patient came to spinal unit for routine ultrasound examination of urinary tract. Ultrasound revealed normal appearance of the kidneys, with no renal calculi or hydronephrosis; balloon of Foley catheter was not seen within the bladder. Urinary catheter had been misplaced, with catheter balloon in the urethra and not within the urinary bladder. Urethral catheter was removed and a size 14 French Foley catheter was inserted by a senior and experienced health professional. The patient was informed about incorrect placement of urethral catheter. We discussed with the patient precautions to be observed while inserting a catheter in order to ensure that the catheter was positioned correctly within the urinary bladder. He was advised to get transurethral catheterisations by senior health professionals. Follow-up ultrasound scan revealed the balloon of the catheter located in the bladder lumen which was otherwise empty.

### Case 3

**Fig. 2:**
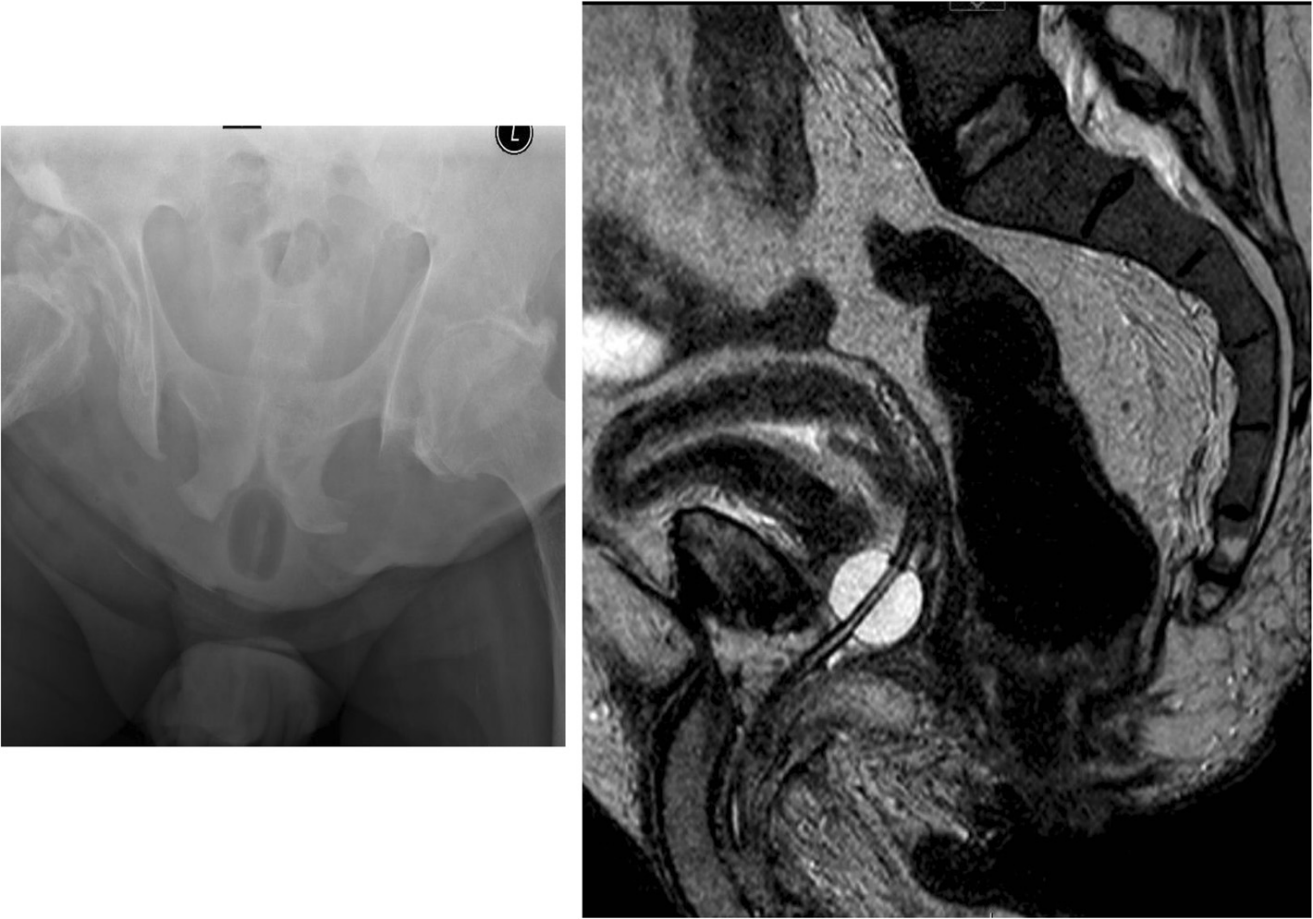
Case 3. Left panel: X-ray of pelvis taken after injecting air through the balloon channel of Foley catheter revealed that the balloon was located in urethra, well below ischiopubic ramus. Right panel: MRI of pelvis sagittal T-2 weighted image revealed balloon of Foley catheter in proximal urethra

A 35-years-old, British Caucasian male, sustained complete paraplegia at T-11 level in 1982, as a result of road traffic accident. Neuropathic bladder was drained by indwelling urethral catheter. In 2012, urethral catheter was changed by a community health professional. There was considerable bleeding per urethra and urine was dark red. He developed rigors and received Trimethoprim 200 mg twice a day for seven days. In 2014, this patient came to spinal unit with the history of bypassing; no urine was going down the catheter. The precise location of the tip of catheter and Foley balloon needed to be checked. It was not possible to do CT or Ultrasound scan on that day. The catheter did not have radio-opaque marking. The patient was allergic to iodine; therefore, 5 ml of air was injected into Foley balloon and X-ray of pelvis was taken. This X-ray revealed the balloon of Foley catheter in urethra (Fig. 2 Left panel). Urethral catheter was removed and another catheter was inserted ensuring that the catheter was located within the bladder. Subsequently, urethral catheter was changed in the community. This patient developed skin breakdown and a cavity in the perineum. Urine was dripping continuously into the open cavity. The balloon of Foley catheter could be seen in the base of the cavity. MRI of pelvis revealed balloon of Foley catheter in the urethra (Fig. 2 Right panel). Suprapubic cystostomy was performed using Seldinger technique (Medi Plus, Unit 7, The Gateway Centre, Coronation Road, Cressex Business Park, High Wycombe HP12 3SU, UK). Following suprapubic cystostomy, the catheter has been draining. The cavity in perineum had shrunk considerably in size. Urine leak continued albeit to markedly lesser extent.

### Case 4

**Fig. 3:**
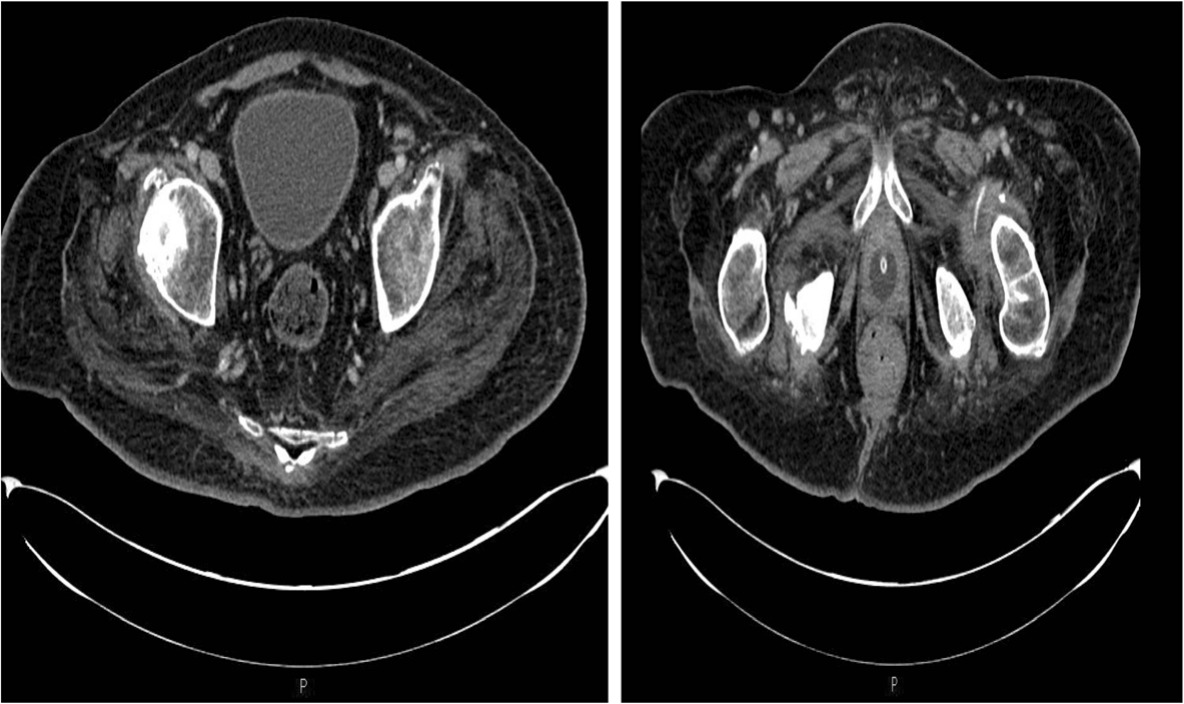
Case 4. Left panel: Axial section of CT of abdomen and pelvis revealed full bladder; no catheter was seen in the lumen of urinary bladder. Right panel: Axial section of CT of pelvis revealed balloon of Foley catheter in the proximal urethra within prostate

A 30-year-old British Caucasian male sustained C-4 tetraplegia in 1988. In 1994, when the patient used the sacral stimulator, penis retracted and penile sheath came off; therefore, he decided to have indwelling urethral catheter In 1997, after routine change of urethral catheter, the catheter did not drain urine; he was taken to Accident and Emergency. When the balloon of Foley catheter was deflated, there was profuse bleeding per urethra. A stiff catheter was inserted. During the next change of catheter, Foley catheter was inserted per urethra and the balloon was inflated; but no urine drained. This patient was taken to Accident and Emergency once again; the balloon was deflated. There was bleeding per urethra. Flexible cystoscopy revealed dilated bulbous urethra, which indicated that the balloon of Foley catheter had been inflated in the urethra. This patient wished to continue with urethral catheter drainage. In 2011, a student nurse inflated the balloon of Foley catheter in urethra; this patient passed blood per urethra and through the catheter. He started getting increased spasms and sweating. A nurse tried to insert a catheter but there was bleeding. Then a doctor tried to insert a catheter per urethra but catheterisation was not possible. Another doctor tried to insert a catheter and again there was bleeding. This patient was taken to theatre; cystoscopy was done and a catheter was inserted per urethra. When the catheter was changed next time, this patient started getting lot of sweating; his spasms got worse. He underwent computed tomography of abdomen to evaluate his bowels. CT revealed distended bladder (Fig. 3 Left panel). The catheter balloon was inflated within the prostatic urethra (Fig. 3 Right panel), which would be the cause for incomplete drainage of urinary bladder. Flexible cystoscopy was performed. There was no false passage. But, prostate-membranous urethra was dilated, which indicated that the balloon of Foley catheter had been inflated in the urethra on past occasions. A 16 French Foley catheter was inserted over a 0.032” guide wire. Subsequently, urethral catheter was changed over a guide wire. Patient noticed a decrease in sweating and severity of spasms.

### Case 5

**Fig. 4:**
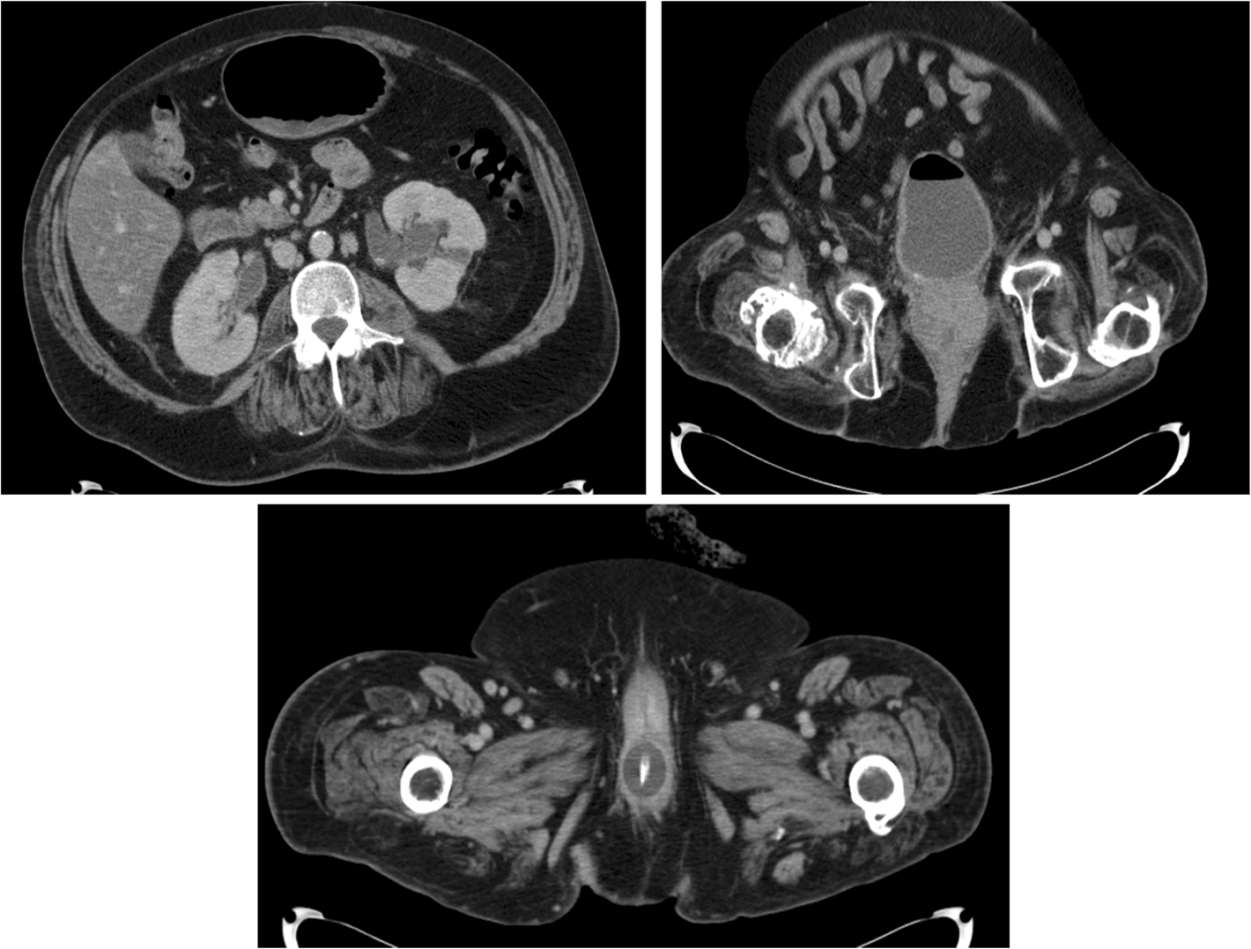
Case 5. Top Left panel: CT abdomen axial section revealed moderate left hydronephrosis and hydroureter, and mild right hydronephrosis and hydroureter. Top Right panel: CT of abdomen and pelvis, axial section: Urethral catheter was not present within the bladder. Bladder was of small capacity and contained urine and gas. Bottom panel: CT pelvis, axial section revealed balloon of Foley catheter in the membranous urethra

A 16-year-old British male was knocked down by a car in 1991. This patient developed T-6 complete paraplegia. He has been managing his bladder by transurethral catheter. Seventeen years after spinal cord injury, urethral catheterisation was difficult; cystoscopy revealed false passage in urethra. A Foley catheter was inserted over a guide-wire. Later, transurethral catheter was pulled out accidentally. Since then urethral catheterisation was performed with flexible cystoscopy and a guide-wire. In 2013, ultrasound of urinary tract revealed small calculi right kidney, largest at the lower pole 6 mm in diameter. No hydronephrosis. Bladder was catheterised and empty. In 2014, ultrasound scan showed no renal calculi; no hydronephrosis. In 2015, he developed malaise, lower abdominal pain, back ache and nausea. He was treated for urine infection. CT abdomen revealed moderate left hydronephrosis and hydroureter, and mild right hydronephrosis and hydroureter down to the bladder (Fig. 4 Top Left panel). Transurethral catheter was not seen in the urinary bladder (Fig. 4 Top Right panel); tip of catheter was present in urethra; Foley balloon was seen in the membranous urethra (Fig. 4 Bottom panel). Most likely, the catheter has been pulled inadvertently. Flexible cystoscopy was performed and a size 16 French Foley catheter with a 20 ml balloon was inserted. Bypassing of catheter and urine leak decreased after inserting a 20 ml balloon Foley catheter per urethra. Subsequently, open suprapubic cystostomy was performed as percutaneous access to urinary bladder was unsuccessful because of his body status and small capacity urinary bladder.

## Discussion

### Risk factors for intra-urethral Foley catheter balloon inflation in spinal cord-injured patients

- Lack of sensation in urethra as a result of spinal cord injury: when the balloon is inflated in urethra, a spinal cord injury patient may not complain of pain or discomfort; so the health professional may not realise that catheterisation has gone wrong.
- Trauma to urethra during previous catheterisations resulting in urethral false passage
- Spasm of pelvic floor muscles and urethral sphincter which prevent the catheter from entering the bladder and leads to curling of Foley catheter in urethra
- Altered anatomy of lower urinary tract due to surgery in the past e.g. bladder neck resection, sphincterotomy.
- Once the balloon of Foley catheter is inflated in urethra, the prostate-membranous urethra becomes dilated; during subsequent catheterisations, the tip of catheter gets doubled back in urethra instead of going straight into the bladder; thus the problem is likely to recur because of chronic dilatation of prostate-membranous urethra.
- Another reason is the habit of using spinal cord injury patients, especially elderly persons, for “practising clinical skills” by student nurses and trainee doctors, be it administration of intramuscular injection, or transurethral catheterisation. Unless this custom is halted, spinal cord injury patients will continue to suffer from iatrogenic complications and receive substandard clinical care [2].

### Clinical features of intra-urethral Foley catheter balloon inflation in spinal cord-injured patients

If a spinal cord injury patient develops bypassing, symptoms of autonomic dysreflexia (headache, profuse sweating and flushing of the skin above the level of the lesion, goose bumps below the level of the lesion, blurred vision, nasal congestion) or increased spasms following transurethral catheterisation, incorrect placement of urethral catheter should be suspected.

Symptoms manifested by the five patients in this case series were:

#### Acute

- Patient number 1 developed bypassing, kidney pain and became unwell.
- Patient number 4 developed urethral bleeding, increased spasms and sweating following change of urethral catheter.
- Patient number 5 developed urine infection and CT revealed bilateral hydronephrosis and hydroureter.

#### Chronic

- Patient number 2, in whom the Foley catheter balloon had been inflated in urethra for weeks, remained asymptomatic. Incorrect placement of urethral catheter was detected during routine ultrasound scan of urinary tract.
- In patient number 3, recurrent inflation of the Foley balloon in urethra resulted in erosion of urethra, breakdown of the skin in perineum and urinary fistula.

The wide spectrum of symptoms of intra-urethral inflation of Foley catheter balloon inflation is summarised below:

Acute (symptoms occurring soon after inflating the Foley balloon in urethra)

i. Bypassing and unsatisfactory drainage through the catheter
ii. Lower abdominal pain, kidney pain
iii. Clinical features of autonomic dysreflexia commonly manifesting as sweating, headache and increased spasms

Chronic (symptoms occurring when the Foley balloon remains inflated in urethra for some time)

i. Urine infection and urosepsis
ii. Bilateral hydronephrosis and hydroureter leading to kidney injury
iii. Erosion of urethra, urethral fistula
iv. Skin breakdown in perineum where the Foley balloon had been kept inflated for prolonged period.

### Diagnosis of intra-urethral Foley catheter balloon inflation in spinal cord-injured patients

When a spinal cord injury patient with indwelling urethral catheter develops bypassing or symptoms of autonomic dysreflexia after transurethral catheterisation, incorrect placement of catheter should be considered. Irrigating the catheter for isovolumetric return and physical examination assessing the length of exposed catheter are often sufficient to diagnose the problem. If the balloon of a Foley catheter is inflated in urethra, an excessive length of catheter will remain outside the penis. This sign is termed “long catheter sign” [3]. If initial bedside assessment is inconclusive, imaging studies should be carried out to locate the position of Foley balloon and tip of catheter. Imaging studies are useful to document what has happened. Ultrasound scan may not be technically satisfactory in some patients because of body habitus; in some others, it may not be possible to see the Foley catheter balloon because urinary bladder is empty or under-filled. CT images showing the Foley catheter balloon inflated in urethra helps to establish the diagnosis; aids greatly in teaching and learning; provides incontrovertible evidence for subsequent case discussion e.g. root cause analysis of this serious adverse event; retraining the involved health professional, dealing with patient complaint and litigation claims; and most importantly identifying the patient as a high-risk case for recurrence of this complication. While carrying out ultrasound or CT scan of urinary tract in patients with urethral catheter drainage, proximal urethra should be scanned if Foley balloon is not visible inside the urinary bladder. Certainly CT scan and MRI should not be routinely utilised to confirm catheter placement as these tests are costly.

### Prevention of intra-urethral Foley catheter balloon inflation in spinal cord-injured patients

i. **Education and Training**
  Education and training of doctors on proper insertion of catheter is important to prevent misplacement of transurethral catheter. Health professionals should adhere to the technique of proper insertion of transurethral catheter in spinal cord injury patients at all times: adequate lubrication, avoid use of size 12CH in difficult cases due to risk of curling of the catheter, insert catheter to the hub, inflate balloon without resistance (an experienced person should be able to tell if the balloon is inflating properly), pulling the catheter back to feel it catch on the bladder neck after balloon inflation, and flushing the catheter with 50 ml of 0.9 % sodium chloride is a quick and easy test to help detect problems (i.e. if it bypasses right out the urethra, or won’t inject, the catheter is probably not positioned correctly.) Urethral trauma from inadvertent inflation of catheter balloon in the urethra during catheterization may be prevented by the use of a safety syringe when this syringe becomes available for routine use [4].
ii. **Transurethral catheterisaion should be performed by experienced health professionals in high risk patients**
  Once the balloon of Foley catheter is inflated in urethra, during subsequent catheterisations, the problem is likely to recur because of chronic dilatation of urethra and sphincter spasm. Therefore, urethral catheterisation should be performed in such high risk patients by senior, experienced health professionals both in the hospital and in the community in order to prevent recurrence of the same complication.
iii. **Choice of Foley catheter balloon size to prevent this complication**
  Depending on the anatomy of urethra and bladder neck in a spinal cord injury patient, appropriate Foley balloon size should be used for transurethral catheterisation. For example, in a patient with wide open bladder neck, a 20 ml balloon Foley catheter is preferable to a standard 10 ml balloon catheter in order to prevent the catheter from slipping into urethra. In case 1, the bladder neck and prostatic urethra became dilated as a result of repeated episodes of inflating Foley balloon in urethra. After insertion of a size 20 ml balloon Foley catheter, transurethral catheter did not slip into urethra.
iv. **Avoid transurethral catheter drainage**
  Intra-urethral Foley catheter balloon inflation can be prevented if urethral catheter drainage is avoided altogether: e.g. by carrying out intermittent catheterisations either by the patient or by carers. Suprapubic cystostomy will help to prevent complications of transurethral catheterisation, but suprapubic cystostomy may lead to different set of problems albeit rarely [5–7].

## Conclusions and Take home message

1. Spinal cord injury patients are at increased risk for inflation of Foley catheter balloon in urethra because of lack of sensation in urethra, urethral sphincter spasm, and previous urethral trauma resulting in false passage.
2. Education and training of doctors and nurses in proper technique of catheterisation in spinal cord injury patients is vital to prevent inflation of Foley catheter balloon in urethra.
3. If a spinal cord injury patient develops bypassing or symptoms of autonomic dysreflexia following transurethral catheterisation, incorrect placement of urethral catheter should be suspected.
4. A simple catheter irrigation and physical examination assessing the length of exposed catheter are often sufficient to diagnose the problem. If initial bedside assessment is inconclusive, imaging studies should be carried out to locate the position of Foley balloon and tip of catheter. Imaging studies are useful to document what has happened.
5. Intra-urethral Foley catheter balloon inflation can be prevented if transurethral catheter drainage is avoided altogether.

## Consent for publication

All patients gave consent for publication of the Case report and any accompanying images.
